# Immunotherapy in Non-Small Cell Lung Cancer With Actionable Mutations Other Than *EGFR*

**DOI:** 10.3389/fonc.2021.750657

**Published:** 2021-12-01

**Authors:** Karan Seegobin, Umair Majeed, Nathaniel Wiest, Rami Manochakian, Yanyan Lou, Yujie Zhao

**Affiliations:** ^1^ Division of Hematology and Oncology, Mayo Clinic, Jacksonville, FL, United States; ^2^ Department of Medicine, Mayo Clinic, Jacksonville, FL, United States

**Keywords:** targeted mutations, immunotherapy, *c-MET*, *RET*, *BRAF*, *ROS-1*, *ALK*, *NTRK*

## Abstract

While first line targeted therapies are the current standard of care treatment for non-small cell lung cancer (NSCLC) with actionable mutations, the cancer cells inevitably acquire resistance to these agents over time. Immune check-point inhibitors (ICIs) have improved the outcomes of metastatic NSCLC, however, its efficacy in those with targetable drivers is largely unknown. In this manuscript, we reviewed the published data on ICI therapies in NSCLC with *ALK*, *ROS1*, *BRAF*, *c-MET*, *RET*, *NTRK*, *KRAS*, and *HER2 (ERBB2)* alterations. We found that the objective response rates (ORRs) associated with ICI treatments in lung cancers harboring the *BRAF* (0–54%), *c-MET* (12–49%), and *KRAS* (18.7-66.7%) alterations were comparable to non-mutant NSCLC, whereas the ORRs in *RET* fusion NSCLC (less than10% in all studies but one) and *ALK* fusion NSCLC (0%) were relatively low. The ORRs reported in small numbers of patients and studies of *ROS1* fusion, *NTRK* fusion, and *HER 2* mutant NSCLC were 0–17%, 50% and 7–23%, respectively, making the efficacy of ICIs in these groups of patients less clear. In most studies, no significant correlation between treatment outcome and PD-L1 expression or tumor mutation burden (TMB) was identified, and how to select patients with NSCLC harboring actionable mutations who will likely benefit from ICI treatment remains unknown.

## Introduction

NSCLC accounts for 85% of all lung cancers, with lung adenocarcinoma being the major subtype (1). Platinum-based combination chemotherapy is the historical first-line standard of care for patients with advanced NSCLC who have no actionable mutations (2). The introduction of ICIs, such as anti-programmed cell death protein ligand 1 (anti-PD-L1) and anti-programmed cell death protein 1 (anti-PD-1) antibodies, as well as the anti-cytotoxic T-lymphocyte-associated protein 4 (anti-CTLA-4) antibody, have revolutionized the treatment of NSCLC, and is typically offered with or without chemotherapy in the front-line setting for incurable NSCLC that does not have any actionable mutations (2). A number of actionable genetic alterations have been identified in NCSLC, including *ALK*, *ROS1*, *c-MET*, *RET*, *NTRK*, *BRAF* V600E, *KRAS*, and *ERBB2 (HER2)* (3–11). MET, RET, HER2, ALK, NTRK, and ROS-1 are receptor tyrosine kinases; BRAF is a serine/threonine kinase mediating cellular signal from RAS to MEK1/2; KRAS is a RAS protein which functions as a GDP–GTP-regulated binary on-off switch. While *c-MET*, *BRAF* and *KRAS* altered NSCLC may develop in both smokers and non-smokers, *ALK*, *ROS1*, *RET*, *NTRK*, and *HER2* alerted NSCLC tend to occur in non-smokers. In patients with actionable driver mutations, namely, *EGFR*, *ALK*, *BRAF*V600E, *RET*, *c-MET*, *NTRK* or *ROS1* alterations, the standard of care is to treat with a Food and Drug Administration (FDA) approved targeted agent, which typically can achieve ORRs of 60–80% in treatment naive patients (2, 12). After targeted therapies are exhausted in these patients, systemic therapy with chemotherapy is typically available for them. While incorporating immunotherapy in the regimen is a standard of care option for them, the efficacy of immunotherapy in those with actionable mutations remains poorly defined due to the limited numbers of these patients included in the randomized prospective trials. In addition to the genetic alterations for which targeted therapies have been approved by FDA in the first line setting in NSCLC, *KRAS G12C* has a targeted agent that was approved recently in the beyond first-line setting. Moreover, *HER2* mutations have emerged as new therapeutic targets with promising therapeutic agents in development. The efficacy of ICI in the *KRAS* G12C or *HER2* mutant NSCLC is also of great clinical interest.

In this modern era with a booming number of treatment options for NSCLC and continued improvement in survival, further guidance is needed on what to expect from the use of immunotherapy in those with these genetic abnormalities. The goal of this review is to add valuable information on the use of immunotherapy in NSCLC with actionable alterations in genes including *ALK*, *ROS1*, *BRAF*, *c-MET*, *RET*, *NTRK*, *KRAS*, and *HER2*. Epidermal growth factor receptor (*EGFR*) mutations are not included in this review as they are included in another manuscript by our group which was submitted separately. In this review, we showed that the sensitivity to ICIs can be heterogenous and differs according to the driver alteration considered. *ALK* and *RET* fusions were found to be associated with low responses to ICI while *BRAF*, *KRAS*, and *c-MET* alterations were associated with responses that were comparable to non-mutant NSCLC, and PD-L1 positive *KRAS* mutant NSCLC may be associated with better outcome when treated with ICI monotherapy as suggested by two retrospective studies. The responses to ICIs are less clear in *HER2*, *ROS1* or *NTRK* altered NSCLCs due to low patient numbers. While an association between PD-L1 expression level or TMB and the responses to ICI has not been consistently observed across all driver alterations, the overall lack of response to ICI treatment appeared to be more common among NSCLC with driver alterations that are typically associated with non-smokers, raising the question whether the absence of tobacco exposure may predict the lack of benefit from ICI treatment. Moreover, the emerging data in the role of co-mutations in response to ICI had also shed a light in the potential underlining mechanism of resistance to ICI, and particularly in the presence of *KRAS* mutation, co-mutations in *TP53*, *STK11*, and *KEAP1* have been found to modulate the response to ICIs in several studies (13–15).

## ALK

Anaplastic lymphoma kinase (*ALK*), a member of the insulin receptor tyrosine kinase family, has been identified as a fusion partner of nearly 30 different proteins in oncogenic signaling in many different cancer types (3). While there are now over 20 *ALK* fusion partners identified in NSCLC, *EML4* represents the most common fusion partner with 29–33% of gene fusions identified to date (16, 17). The fusion of the 5′ end partner *EML4* to the coding region of the intracellular tyrosine kinase domain of *ALK* leads to aberrant expression of the ALK fusions in the cytoplasm. The domains in the partner proteins also promote dimerization and oligomerization of the fusion proteins, leading to constitutive activation of *ALK* kinase and its downstream signaling pathways including RAS–mitogen-activated protein kinase, phosphoinositide 3-kinase-AKT, and JAK-STAT pathways. This subsequently results in uncontrolled cellular proliferation and promotes survival (3, 18). *ALK* fusions are seen in 3–5% of NSCLC patients and are more common among the following groups: no prior smoking history, adenocarcinoma histology, younger age, female gender, and tumors with wild type *EGFR* and *KRAS* (16, 19–21). Several *ALK* inhibitors have been approved by the FDA for metastatic NSCLC, including crizotinib, brigatinib, alectinib, lorlatinib and ceritinib (22–29). The data on the efficacy of ICIs in the *ALK* fusion positive NSCLC has been scarce. It has been postulated that EML4-ALK oncoprotein can upregulate the PD-L1 expression in lung cancer cells. In one report of 100 patients, fifty patients (50.0%) were PD-L1 negative, 34 patients (34.0%) were PD-L1 low expression (tumor proportion score [TPS] 1–50%), and 16 patients (16.0%) had a strong PD-L1 expression (TPS ≥ 50%) (30). Despite the expression of PDL1 in these tumors, the overall response to ICIs in the *ALK* fusion positive population has been disappointing except in one study (**Table 1**).

**Table 1:** Efficacy of ICIs in NSCLS with *ALK* mutations.

Reference	Characteristics	ORR, %	mPFS, months	mOS, months since start of ICI
Mazieres J., et al. (31)	*ALK* (n=23)	0	2.5	17
Gainor JF.,et al. (32)	*ALK* (n=6)	0		
Jahanzeb M., et al. (33)	*ALK* (n=83)		2.34	
Gadgeel SM. et al. (34)	*ALK (n=7)*	28.6%	2.9	2.9

ICIs, immune checkpoint inhibitors; NSCLC, non-small cell lung cancer; ORR, overall response rate; mPFS-median progression-free survival; mOS, median overall survival.

Although small numbers of patients with *ALK* fusion NSCLC were included in the randomized phase 3 CheckMate 057 and KEYNOTE-010 studies comparing ICI versus docetaxel in previously treated NSCLC patient population, the outcomes in this specific population were not reported (35, 36).

In a retrospective study using the IMMUNOTARGET registry which included 551 patients receiving ICI monotherapy for advanced NSCLC with at least one oncogenic driver alteration, 23 patients with *ALK* fusion NSCLC were identified (31). The objective response rate to ICI treatment was 0%. The Median PFS was 2.5 (1.5; 3.7) months. The median OS from start of ICI therapy was 17.0 (3.6; NR) months. Among the 10 patients with available PD-L1 status, the median percentage of cells expressing PD-L1 was 7.5% (**Table 1**).

In a retrospective study conducted at the Massachusetts General Hospital, the ORR to ICI treatment among patients with *EGFR* mutations or *ALK* rearrangements was only 1/28 (3.6%) while the ORR among *EGFR WT/ALK*-negative patients was 7/30 (23.3%) (P = 0.053) (32). Since the lone partial response was seen in an *EGFR*-mutant patient, it appeared that none of the six *ALK* fusion NSCLC patients had a response (**Table 1**).

In the randomized Impower130 study, atezolizumab plus chemotherapy (Nab-Paclitaxel and Carboplatin) did not show improved overall survival versus chemotherapy alone in the subset of 44 patients with *EGFR* or *ALK* genomic alterations in the first line setting (37). However, in the Impower150 study, the addition of Atezolizumab to Bevacizumab, Carboplatin, and Paclitaxel improved the median PFS for patients with *EGFR* or *ALK* genomic alteration whose diseases had progressed on TKI or who were unable to tolerate TKI (median, 8.3 months vs. 6.8 months; stratified hazard ratio, 0.61; 95% CI, 0.52 to 0.72). Of note, 34 patients with *ALK* fusion and 80 patients with *EGFR* mutant nonsquamous metastatic lung cancer were included in this study, and information on the benefit of atezolizumab in *ALK* fusion NSCLC was not reported separately (38). In another report of 83 patients with *ALK* mutation treated with ICI, a mPFS of 2.34 months was reported (33).

A recent prospective multicenter trial presented at the World Conference on Lung Cancer evaluated pembrolizumab and chemotherapy in the setting of recurrent *EGFR*/*ALK*-positive NSCLC. The study enrolled a total of 33 patients, including 26 *EGFR* mutant NSCLC and seven *ALK* fusion positive NSCLC patients. Most of the patients had one prior targeted therapy. No more than one prior line of platinum-based chemotherapy for advanced NSCLC was allowed. In those with ALK-positive tumors, the ORR was seen in 2/7 (28.6%), and the mPFS and mOS were both 2.9 months, suggesting lack of benefit of ICI in this group of patients (34).

## BRAF

*BRAF* is a serine/threonine kinase mediating cellular signal from RAS to MEK1/2, and BRAF activation can result in phosphorylation and activation of extracellular signal-regulated kinase (ERK)1/2, leading to cell survival and proliferation (4). *BRAF* mutations are found in 1.5–3.5% of NSCLC with V600E accounting for approximately half of those mutations (39). Besides adenocarcinoma, *BRAF* mutations have been reported in sarcomatoid carcinomas, large-cell neuroendocrine carcinomas, and squamous cell lung cancer (40, 41). *BRAF* mutations can occur in both smokers and non-smokers (42). Selective kinase inhibitors have been recommended for the first-line and second-line treatments of *BRAF* V600E mutant advanced NSCLC with a reported ORR as high as 64% in this group of patients (39). The outcomes associated with ICIs in this population have been studies in multiple retrospective analyses (**Table 2**). Although the data vary significantly among different studies, responses to ICI have been seen in most of the studies.

**Table 2:** Efficacy of ICIs in NSCLS with *BRAF* mutations.

Reference	Characteristics		ORR, %	mPFS, months	mOS, months since start of ICI
Dudnik E., et al. (43)	Total (n=22)		28		
	mutation type	V600E (n=12)	25	3.7	Not reached (median follow-up of 5.5 months)
		nonV600E (n=10)	33	4.1	Not reached (median follow-up of 5.5 months)
	PD-L1 expression	PD-1L ≥50%	36	5.3	
		PDL-1 0-49%	14	2.2	
Rihawi K., et al. (44)	*BRAF*, 2^nd^ line immunotherapy (n=11)		9		10.3
Tan I., et al. (45)	*BRAF*, 1st line immunotherapy (n=3)			0.17, 1.4, and 4.4 for each patient respectively	0.17, 6.8, and 7.5 for each patient respectively
	*BRAF*, 2^nd^ line immunotherapy (n=8)			2.5	
	*BRAF*, 1^st^ line chemoimmunotherapy (n=2)			1.5 and 2.1 for each patient respectively	6.6 and 5.6 for each patient respectively
Mazieres J., et al. (31)	*BRAF* (n=43)		24.3	3.1 (1^st^-3^rd^ line ICI)	20.3
				2.7 (>3^rd^ line)	
Dudnik E., et al. (46)	*BRAF* V600E (n=5)		25	1.5	NR (not reached)
	*BRAF* non-V600*E* (n=5)		20	2.6	NR (not reached)
Guisier F., et al. (47)	*BRAF* V600E (n=26)		26.1%	5.3	22.5
	*BRAF* non-V600E (n=18)		35.3%	4.9	12
Mu Y., et al. (48)	*BRAF*(n=9)		25%	3.0	

ICIs, immune checkpoint inhibitors; NSCLC, non-small cell lung cancer; ORR, overall response rate; mPFS-median progression-free survival; mOS, median overall survival.

In a retrospective study including seven participating Israeli cancer centers reported by Dudnik et al., PD-L1 expression level, tumor mutational burden (TMB), and microsatellite instability status were assessed in both *BRAF* V600E and non-V600E *BRAF* mutation positive NSCLC, and the outcome with ICI treatment was reported (43). High (≥50%) PD-L1 expression was found to be more common in the *non-BRAF* V600E mutant group than the V600E *BRAF* mutant group (50% vs 42%, *p* = 0.05). No MSI-H was found in both groups, and the median TMB was 5 (1–42) muts/Mb and 11 (7–14) muts/Mb in the *BRAF* V600E and the non-V600E *BRAF* mutant groups, respectively. ICI therapy was associated with ORRs of 25 and 33% in the *BRAF* V600E and the non-V600E *BRAF* mutant positive groups, respectively (p = 1.0) (**Table 2**). Among the six patients with high PD-L1 and *BRAF* V600E mutant NSCLC, two patients had major tumor shrinkage while two other patients had hyperprogression (43).

Among the 1,588 advanced non-squamous NSCLC patients enrolled in the Italian Expanded Access Program of second line nivolumab, 210 patients were assessed for *BRAF* mutations, and 11 patients (5%) were found to be positive. Median OS was comparable among different groups, and was found to be 11.0 months (range: 9.8 to 12.2 months), 11.2 months (range: 9.2 to 13.2 months) and 10.3 months (range: 2.1 to 18.5 months) in the population with unknown *BRAF* status, *BRAF* wild-type subgroup, and *BRAF* mutated subgroup, respectively (44) (**Table 2**).

A retrospective study was conducted to evaluate the clinical response to immunotherapy and chemotherapy among 31 patients with *BRAF* mutant metastatic NSCLC treated at the Duke University Hospital (45). PD-L1 expression information was only available for 11 patients. PD-L1 expression levels ranged from 0 to 90%, with six patients with PD-L1 expression levels greater than 50%. TMB was only available on five patients, ranging from 3 to 18 mutations/Mb. The median PFS in patients who received first-line chemotherapy was 6.4 months (95% CI, 2.3 to 13.0) while the PFS of each of the three patients who received first-line immunotherapy was 0.17, 1.4, and 4.4 months. The median OS in patients who received first-line chemotherapy was 18.4 months (95% CI, 7.4 to 28.6), and the OS of each of the three patients who received first-line immunotherapy was 0.17, 6.8, and 7.5 months (**Table 2**).

In the retrospective study using the IMMUNOTARGET registry, among the 43 patients with *BRAF* mutations, PFS was significantly higher in smokers than never smokers (4.1 versus 1.9 months, *P* = 0.03), however shorter in the V600E subgroup (1.8 months) compared with other *BRAF* mutations (4.1 months, *P* = 0.20) (31) (**Table 2**). The ORR was 24.3%. Among the nine patients with available PD-L1 status, the median percentage of cells expressing PD-L1 was 50%.

In the IMAD2 (GFPC 01-2018), a retrospective study that included 21 centers in France reported by Guisier et al., 44 ICI-treated *BRAF* mutant (*BRAF* V600E, n = 26; *BRAF* non-V600E, n = 18) NSCLC patients were identified (47). Most of the patients received ICI in the beyond-first line setting. Response rates for *BRAF-*V600E- and *BRAF-*non-V600E- mutant NSCLC were 26 and 35%, respectively. The median DORs to ICI were NR (95% CI 12.6–NR) and 13.1 months (95% CI 7.6–NR) in the *BRAF-*V600E- and *BRAF-*non-V600E groups. The PFS in the *BRAF-*V600E- and *BRAF-*non-V600E groups were 5.3 months (95% CI 2.1–NR) and 4.9 months (95% CI 2.3–NR), and the OS in the *BRAF-*V600E- and *BRAF-*non-V600E groups were 22.5 months (95% CI 8.3–NR) and 12 months (95% CI 6.8–NR). The 12-month OS in the *BRAF-*V600E- and *BRAF-*non-V600E- groups were 53.4 and 44%, respectively (**Table 2**).

In a cohort of 10 patients with tumors harboring *BRAF* mutations (*BRAF* V600E, n−5; *BRAF* non-V600E, n−5) who received ICI treatment, ORR of 25% (1/4) and 20% (1/5) were seen in patients with *BRAF* V600E mutation and *BRAF* non-V600E mutation, respectively (46). Median PFS comprised 1.5 months (95% CI, 1.2–8.3) in patients with *BRAF* V600E mutation and 2.6 months (95% CI, 2.0–4.2) in patients with *BRAF* non-V600E mutation. Median OS was not reached in patients with *BRAF V600E* mutation (95% CI, 1.2–NR) or *BRAF* non-V600E mutation (95% CI, 2.3–NR) (46) (**Table 2**). Among patients with known PD-L1 TPS, TPS high (≥50%) was seen in 25 and 60% of the *BRAF* V600E- and non*-BRAF* V600E-mutant NSCLC cases, respectively. TMB high (≥10 mut/Mb) was seen in 3 and 1% of the *BRAF* V600E- and non-*BRAF* V600E-mutant NSCLC cases, respectively. No MSI-H/I was seen.

In another report of nine patients with *BRAF* (*BRAF* V600E, n−6; *BRAF* non-V600E, n−3) who received ICI with chemotherapy or antiangiogenic treatment, the ORR was 25% and mPFS was three months (95%CI 2.9, 3.1) (48).

## MET

MET is a proto-oncogene receptor tyrosine kinase that mediates cell proliferation, survival, and metastasis (5). Recurrent somatic splice site alterations at *MET exon 14 (METex14)* can result in exon skipping, decreased MET degradation, and MET activation. *METex14* is involved in cancer through promoting angiogenesis, cell migration, and invasion (49, 50). *METex14* occurs in 3–4% of lung cancers and 8–30% of sarcomatoid lung cancers (51, 52). The occurrence of *METex14* appears to be independent of smoking status (53). FDA has granted accelerated approval to capmatinib and tepotinib for adult patients with metastatic NSCLC whose tumors have a mutation that leads to *METex14* alterations (54, 55).

In a retrospective study that included 147 patients with *METex14* lung cancers, PD-L1 expression of ≥50% was identified in 41% of 111 evaluable tumor samples. The median TMB of *METex14* lung cancers was lower than that of unselected non-small-cell lung cancers (NSCLCs). In 24 response-evaluable patients, the ORR was 17% (95% CI 6 to 36%) and the median PFS was 1.9 months (95% CI 1.7–2.7). Responses were not associated with PD-L1 expression ≥50% or high TMB (12) (**Table 3**).

**Table 3:** Efficacy of ICIs in NSCLS with *c-MET* mutations.

Reference	Characteristics	ORR, %	mPFS, months	mOS, months since start of ICI
Sabari JK. et al. (12)	*cMET exon 14* skipping mutation (n=147)	17	1.9	18.2
Mazieres J., et al. (31)	*cMET exon 14* skipping mutation and *cMET* amplification (n=36)	49	3.4	18.4
Guisier F., et al. (47)	*cMET* mutant (n=30)	36	4.9	13.4
Dudnik- E., et al. (46)	*cMET exon 14* skipping mutation (n=148)	12	4	NR (not reached)
	*cMET* amplification (n=54)	25	4.9	NR (not reached)
Mayenga M., et al. (56)	*cMET exon 14* skipping mutations, 2^nd^ line immunotherapy (n=13)	46.2		

ICIs, immune checkpoint inhibitors; NSCLC, non-small cell lung cancer; ORR, overall response rate; mPFS, median progression-free survival; mOS, median overall survival.

Among the 551 patients in the IMMUNOTARGET registry, 13 patients with *MET* amplification and 23 patients with *METex14* were identified (31). Median OS from ICI initiation of this 36-paitent cohort was 18.4 months (7.0; NR) (31). Progressive disease (PD) was found to be the best response to ICI among 50% of patients, and median PFS was found to be 3.4 months (1.7; 6.2). Long-term responders were seen in 23.4% of patients (**Table 3**). Among the 15 patients with available PD-L1 status, the median percentage of cells expressing PD-L1 was 30%.

In the French retrospective study IMAD2 (GFPC 01-2018), 30 cases of ICI-treated *MET* mutant NSCLC were identified (47). Most patient received ICI in the beyond-first line setting. The response rate for *MET*-altered NSCLC was 36%. The median duration of response (mDOR) was 10.4 months (95% CI 4.6–NR). The mPFS was 4.9 months ((95% CI 2.0–11.4), and the mOS was 13.4 months (95% CI 9.4–NR) (**Table 3**).

In a retrospective study that included eight cases of NSCLC with *METex14* and four cases of NSCLC with *MET* amplification treated with ICI, median PFS with ICI was 4.0 months (95% CI, 2.4–NR) in patients with *METex14* and 4.9 months (95% CI, 2.4–NR) in patients with *MET* amplification (46). ORR comprised 12% (1/8) and 25% (1/4) in patients with *METex14* and *MET* amplification respectively. Median OS with ICI was not reached in patients with *METex14* (95% CI, 4.1–NR) or in patients with *MET* amplification (95% CI, 3.5–NR) (**Table 3**). Among patients with known PD-L1 TPS, TPS high (≥50%) was seen in 67% of the cases. TMB high (≥10 mut/Mb) or MSI-H/I was not seen.

In a case series, among 13 patients with *METex14* NSCLCs treated with ICI, 46.2% (6/13) patients responded to immunotherapy. Six patients had prolonged duration of responses ranging from 18 months (still ongoing) to 49 months (56).

## RET

*RET* is a proto-oncogene receptor tyrosine kinase that binds with the ligand–co-receptor complex of glial cell line-derived neurotrophic factor (GDNF) family ligands (GFLs) and subsequently activates signaling pathways such as RAS/mitogen activated protein kinase (MAPK), RAS/ERK, phosphatidylinositol 3-kinase (PI3K)/AKT, and c-Jun N-terminal kinase (JNK). Aberrant activation of the RET receptor have been associated with multiple endocrine neoplasia 2 (*MEN2*), sporadic medullary thyroid cancer, papillary thyroid carcinoma (PTC), and non-small cell lung cancer (NSCLC) (6, 7). *RET* rearrangements have been identified in 1–3% of NSCLC and were found to have significantly higher frequencies in younger (<60 years of age), female, non-smokers, and adenocarcinoma histology (57–60). *RET* fusion positive NSCLC is usually associated with low PD-L 1 expression (61). Two potent selective RET inhibitors, selpercatinib and pralsetinib, have been approved by the FDA for *RET* fusion-positive NSCLC (62, 63). The activity of ICI in *RET* altered NSCLC has been evaluated in multiple studies, and the benefit of ICI was found to be low in most of the studies (**Table 4**).

**Table 4:** Efficacy of ICIs in NSCLS with *RET* mutations.

Reference	Characteristics	ORR, %	mPFS, months	mOS, months since start of ICI
Guisier F., et al. (47)	*RET* fusion (n=9)	37.5	7.6	NR (not reached)
Lee J., et al. (64)	*RET* fusion (n=13)	7.7	2.1	12.4
Mazieres J., et al. (31)	*RET* fusion (n=16)	6	2.1	21.3
Offin M., et al. (61)	*RET* fusion (n=16)	0	3.4	
Dudnik E., et al. (46)	*RET* fusion (n=4)	0	3	14.9
	*RET* mutation (n=1)	0	6.9	15.3

ICIs, immune checkpoint inhibitors; NSCLC, non-small cell lung cancer; ORR, overall response rate; mPFS, median progression-free survival; mOS, median overall survival.

In the French retrospective study IMAD2 (GFPC 01-2018), nine patients with *RET* translocation NSCLC received ICI, all in the beyond-first line setting. The response rate for *RET*-altered NSCLC was 38%. The mDOR response to ICI was 12.1 months (95% CI 8.4–NR). The median PFS was 7.6 months (2.3–NR), and the median OS was not reached (95% CI 26.8–NR) (47) (**Table 4**).

In a single center retrospective study conducted in Korea, the median progression-free survival for ICI among 13 patients with *RET* fusion-positive NSCLC treated with ICI was 2.1 (95% CI: 1.6–2.6) months, and the ORR was 7.7% (64). The median PFS and OS were 2.1 (95% CI: 1.6–2.6) and 12.4 (95% CI: 2.9–21.8) months, respectively. Among patients with PD-L1 expression 25% and above, 2/5 patients demonstrated stable disease, while the best response in the other three patients was disease progression (**Table 4**). In contrast, the ORR and DCR among 46 patients treated with pemetrexed-based regimens in this study was 63.0 and 91.3%, respectively, and the median PFS was 9.0 (95% CI: 6.9–11.2) months.

Among the 16 patients with *RET* fusion-positive NSCLC in the IMMUNOTARGET registry, the median OS from the start of ICI therapy was 21.3 (3.8; 28.0), and the median PFS was only 2.1 (1.3; 4.7) (31). The rate of any partial or complete response was very low and was 6.3% (1/16) (**Table 4**). Among the six patients with available PD-L1 status, the median percentage of cells expressing PD-L1 was 26%.

In a retrospective study conducted at the Memorial Sloan Kettering Cancer Center, 13 patients with *RET*-rearranged NSCLC treated with ICI were assessed for clinical and/or radiologic response (30). No response to immunotherapy was observed. The median PFS was 3.4 months (95% CI, 2.1 to 5.6 months). No difference in OS between patients with advanced *RET*-rearranged lung cancers who received immunotherapy (n = 16) and those who did not receive immunotherapy (n = 46), (hazard ratio, 1.4 [95% CI, 0.7 to 2.9]; log-rank P = .35) (**Table 4**). Only one patient was found to have PD-L1 expression ≥50%, and the disease of this patient did not respond to ICI. No patient had TMB >10 mut/Mb.

In the single institution retrospective study published by Dudnik et al., four patients with *RET* fusion NSCLC and one patient with *RET* mutant NSCLC were treated with ICI (46). No objective response was observed. Median PFS was 3.0 months (95% CI, 1.9–3.1) in patients with *RET* fusion and 6.9 months in patient with *RET* mutation. Median OS since start of ICP were 14.9 months (95% CI, 7.2–19.7) in patients with *RET* fusion and 15.3 months in patient with *RET* mutation (**Table 4**). Among patients with known PD-L1 TPS, TPS high (≥50%) was seen in 13 and 0% of the *RET* fusion and the *RET* mutant NSCLC cases, respectively. TMB high or MSI-H/I was not seen.

## ROS1

*ROS* proto-oncogene 1 (*ROS1*) belongs to the subfamily of tyrosine kinase insulin receptors (65). *ROS1* fusion can lead to constitutive activation of kinase activity, resulting in increased cell proliferation, survival, and migration due to the upregulation of JAK/STAT, PI3K/AKT, and MAPK/ERK signaling pathways (8). *ROS1* rearrangements account for 1–2% of NSCLC patients (66, 67). This alteration more frequently occurs in adenocarcinoma and in younger patients with no or light smoking history (68, 69).

Seven patients with *ROS1* fusion NSCLC treated with ICI were identified in the IMMUNOTARGET registry (31). The objective response rate to ICI treatment was 17% (**Table 5**).

**Table 5:** Efficacy of ICIs in NSCLS with *ROS-1* mutations.

Reference	Characteristics	ORR, %	mPFS, months	mOS, months since start of ICI
Mazieres J., et al. (31)	*ROS1* (n=7)	17		
Dudnik E., et al. (46)	*ROS 1* (n=1)		0.1	0.1

ICIs, immune checkpoint inhibitors; NSCLC, non-small cell lung cancer; ORR, overall response rate; mPFS, median progression-free survival; mOS, median overall survival.

In the single institution retrospective study published by Dudnik et al., only one patient with *ROS1* fusion NSCLC treated with ICI was identified, and the reported PFS and OS were both 0.1 month (46) (**Table 5**). Among the five patients with available PD-L1 status, the median percentage of cells expressing PD-L1 was 90%.

In the Japanese retrospective study, 15 *ROS1* altered NSCLC cases were identified. High expression of PD-L1 (>50% of tumor cells by 22C3) were observed in 53% cases, however, no response to immunotherapy was observed (70).

## NTRK

The *NTRK* genes (*NTRK1*, *NTRK2* and *NTRK3*) encode tropomyosin receptor kinases (*TRKA*, *TRKB* and *TRKC*) (9). The *TRK* fusion protein leads to constitutive activation of various downstream signal transduction pathways including the PI3k/Akt and RAS/RAF/MAPK pathways, and subsequently causes proliferation of cancer cells (9). Rearrangements including *NTRK1*, *NTRK2*, and *NTRK3* occur in approximately 2–3% of NSCLC patients (10). Selective *TRK* inhibitors, Entrectinib and Larotrectinib, have been approved for patients with *NTRK* fusion-positive solid tumors, including NSCLC (71, 72).

In the single institution retrospective study published by Dudnik et al., two patients with *NTRK* fusion NSCLC were treated with ICI. The objective response rate was 50% (1/2). Median PFS was as not reached (95% CI, 3.2–NR). Median OS since start of ICP not reached (95% CI, NR–NR) (46) (**Table 6**). One patient had PD-L1 TPS ≥50%. No patient had TMB ≥10 muts/Mb.

**Table 6:** Efficacy of ICIs in NSCLS with *NTRK* mutations.

Reference	Characteristics	ORR, %	mPFS, months	mOS, months since start of ICI
Dudnik E., et al. (46)	*NTRK* (n=2)	50%	Not reached	Not reached

ICIs, immune checkpoint inhibitors; NSCLC, non-small cell lung cancer; ORR, overall response rate; mPFS, median progression-free survival; mOS, median overall survival.

## *KRAS* G12C

KRAS is one of the RAS proteins (KRAS4A, KRAS4B, NRAS, and HRAS) which function as GDP–GTP-regulated binary on-off switches and regulate cell survival, cell cycle progression, cell polarity, movement, and nuclear transport by transducing signals from transmembrane receptors to cytoplasmic signaling pathways such as the MAPK pathway (10, 11). It is the most common proto-oncogene identified in NSCLC. *KRAS* mutations occur in 15–25% of lung adenocarcinomas and are more prevalent in smokers than nonsmokers (73, 74). Majority of the *KRAS* mutations in NSCLC occur on exon 2 or 3 (G12, G13, and Q61), with the most frequent being the G12C followed by G12V and G12D (75, 76). Sotorasib has been approved by the FDA for patients with *KRAS* G12C mutant locally advanced or metastatic NSCLC in the beyond the first line setting (72). It is associated with an objective response rate of 37.1% in this group of patients (77). The efficacy of ICIs in KRAS mutant NSCLC has been studied in several retrospective studies, and most of the data support the benefit on ICIs in *KRAS* mutant NSCLC (**Table 7**).

**Table 7:** Efficacy of ICIs in NSCLS with *KRAS* mutations.

Reference	Characteristics	ORR, %	mPFS, months	mOS, months since start of ICI
Mazieres J., et al. (31)	*KRAS* (n=271)	26%	3.2 (1^st^-3^rd^ line ICI)	13.5
			3.1 (>3^rd^ line)	
			G12C: 5.5 G12A: 4.4 G12D: 3.2 G12V: 1.9 G12S: 2.1	
Herbst RS., et al. (78)	Any *KRAS* (n=69), first line immunotherapy	56.7%	12	28
	*KRAS* G12C	66.7%	15	NR
Sun L., et al. (79)	Any *KRAS* (n=573), first line monotherapy or chemoimmunotherapy			21.1 (ICI monotherapy) 20 (chemoimmunotherapy)
Gadgeel SM. et al. (80)	Any *KRAS* (n=89), first line chemoimmunotherapy	40.7%	9	21
	*KRAS* G12C	50%	11	18
West H., et al. (81)	*KRAS (n=80)* (first line chemoimmunotherapy with VEGFR targeted therapy)		8.11	19.81
	With *mutant STK* and/or *mutant KEPA1 (n=34)*		6.03	11.1
	With wild-type *STK* and wild-type *KEPA1 (n=46)*		15.21	26.18
Passiglia F., et al. (82)	*KRAS*, (n=206)	20%	4	11.2
Jeanson A., et al. (83)	*KRAS* (n=162)	18.7%	3.09	14.29

ICIs, immune checkpoint inhibitors; NSCLC, non-small cell lung cancer; ORR, overall response rate; mPFS, median progression free survival; mOS, median overall survival, HR, hazard ratio, CI, confidence interval.

In a retrospective analysis in patients enrolled in the KEYNOTE-042 evaluating pembrolizumab monotherapy vs platinum-based chemotherapy as the first-line therapy among patients with PD-L1-positive (TPS ≥1%) advanced non-squamous histology NSCLC, 301 patients were evaluable by whole-exome sequencing (WES). *KRAS* mutations were found in 69 (23%) patients, among which, 29 (10%) patients were found to have *KRAS* G12C (78). PD-L1 TPS and TMB were found to be higher in patients with *KRAS* mutations than without *KRAS* mutations, although the differences were not significant. The OS associated with pembrolizumab was better than chemotherapy in both the *KRAS* mutant group and *KRAS* G12C subgroup, with the HRs being 0.42 (0.22–0.81) and 0.28 (0.09–0.86), respectively. Conversely, there was no significant OS difference seen between pembrolizumab and chemotherapy in the *KRAS* wild-type patients, and HR was 0.86 (0.63–1.18). A superior PFS was also observed when pembrolizumab was compared with chemotherapy in the *KRAS* mutant patients. The data supported the benefit of single agent pembrolizumab in the PD-1 TPS >1% *KRAS* mutant (including *KRAS* G12C) NSCLC patients, underlining the important role of ICI in the treatment of this group of patients.

The efficacy of ICIs in the first line setting in PD-L1 TPS ≥50% advanced NSCLC was also investigated in a retrospective analysis using the Flatiron Health database (79). Among the1,127 patients with PD-L1 expression of 50% or greater who were treated with either ICI monotherapy or chemoimmunotherapy, 573 (50.8%) had *KRAS* alterations and 554 (49.2%) had wild type *KRAS*. Among the patients treated with ICI monotherapy, a better mOS was seen in the *KRAS* mutant group when compared with the wild-type group (mOS, 21.1 vs 13.6 months; P = .03). Interestingly, this OS advantage was not observed among patients treated with chemoimmunotherapy, and the mOS was 20.0 vs 19.3 months; P = .93 in the *KRAS* mutant and wild type patients. Furthermore, no mOS difference was seen between ICI monotherapy and chemoimmunotherapy in the *KRAS* mutant NSCLC patients (mOS, 21.1 vs 20.0 months; P = .78), suggesting that the use of ICI monotherapy in the PD-L1 TPS ≥50% is an acceptable option in the *KRAS* mutant advanced NSCLC.

The efficacy of chemoimmunotherapy in *KRAS* mutant NSCLC was also analyzed retrospectively in the participants of another randomized trial, the KEYNOTE-189 study of pembrolizumab plus pemetrexed and platinum chemotherapy vs placebo plus chemotherapy as first-line therapy for metastatic non-squamous NSCLC (80). Among the 289 patients who had evaluable WES data, 89 (31%) patients were found to have *KRAS* mutations including *KRAS* G12C, which was found in 37 (13%) patients. As observed in the KEYNOTE-042 study, the higher PD-L1 TPS and TMB tended to be seen with KRAS mutant patients. Although unlike the observation in the KEYNOTE-042, the OS benefit associated with the addition of ICI was only detected in the *KRAS* wild-type patients. PFS improvement associated with the additional of ICI was seen in both the *KRAS* mutant and wild type group but not in the *KRAS* G12C subgroup, which could be related to the small sample number.

In addition to ICI monotherapy and chemoimmunotherapy, the combination of VEGF receptor targeted agent and chemoimmunotherapy represents another first-line treatment option for advanced NSCLC based on the IMpower150 study (84). A *post hoc* analysis evaluated the efficacy outcomes in patients with *KRAS*, *STK11(LKB1)*, and *KEAP1* mutations (81). Among 920 patients included, *KRAS* mutations were found in 80 patients (24.5%), with 39 patients found to have co-occurring mutations in *STK11* and/or *KEAP1*. The addition of ICI improved mOS and PFS in the *KRAS* mutant patients regardless of STK11 and KEAP1 status (**Table 7**), supporting the use of this regimen in *KRAS* mutant NSCLC.

The correlation between STK11/LKB1 genomic alterations and the efficacy of ICI treatment in KRAS mutant NSCLC was also evaluated using the Stand Up To Cancer (SU2C) dataset (13). Unlike the *post hoc* analysis of the IMpower150 study, this study showed that the concurrent *STK11/LKB1* mutation in *KRAS* mutant NSCLC was associated with an inferior ORR to PD-1 blockade when compared with *KRAS* mutation without *STK11/LKB1* mutation and *KRAS* mutation with *P53* mutations groups (7.4, 28.6 and 35.7% (P <0.001)). The details of the ICI therapy in this dataset were not available, and it is unclear whether this group of patients also received angiogenesis targeted agent treatment.

In a systemic review and metanalysis aiming to investigate the predictive clinicopathological characteristics for the relative efficacy of ICIs vs docetaxel in the second-line setting in NSCLCs, the authors analyzed data from five randomized clinical trials involving 3,025 patients (85). ICIs were associated with prolonged overall survival (HR, 0.69; 95% CI, 0.63–0.75; *P* < .001). The survival benefit was also seen among the 148 *KRAS* mutant patients (HR, 0.65; 95% CI, 0.44–0.97; *P* = .03) but not in the 371 *KRAS* wild-type patients (HR, 0.86; 95% CI, 0.67–1.11; P = .24; interaction, P = .24) (85).

The efficacy of ICI in *KRAS* mutant non-squamous NSCLC in the beyond first-line setting was also investigated in patients who received nivolumab in an Italian expanded access program (EAP) study (82). Among the 530 patients evaluated, 206 (39%) had *KRAS* mutations. No significant differences in OS, PFS or ORR were seen between *KRAS* mutant and *KRAS* wild- type patients in this study, supporting that nivolumab should be considered for patients regardless of *KRAS* mutation status. Interestingly, any significantly higher grade and grade 3–4 treatment related adverse events were seen in the *KRAS* mutant group than the wild-type group, although the underlining mechanism for the finding is unknown.

*KRAS* mutant NSCLC was also evaluated in the IMMUNOTARGET study. Two hundred and seventy-one patients treated with ICIs were found to have *KRAS* mutations. An encouraging ORR of 26% was found, and the mPFS and mOS were 3.2 and 13.5months, respectively (31).

In a single instituation retrospective study conducted in France, a total of 162 *KRAS*-mutant advanced NSCLC were identified among the 282 subjects analyzed. No significant difference was seen in ORR, mPFS or mOS between the *KRAS* mutant and the *KRAS* wild-type groups. The ORR, mPFS, and mOS associated with ICI of *KRAS* mutant NSCLC were 18.7%, 3.09 months and 14.29 months. No significant difference in treamtent outcomes was seen among the KRAS mutation subtypes including G12A, G12C, G12D, G12V, and G13C (83).

## HER 2

Human epidermal growth factor 2 (HER2 erbB-2/neu) is one of the four receptor tyrosine kinase members of the human epidermal growth factor receptor family. Upon forming homo- or hetero-dimers with other family members, HER2 becomes activated and signal through the PI3K-AKT and MEK-ERK downstream pathways to activate proliferation (86). In NSCLC, activating *HER2* mutations occur in 2–4% of cases, most commonly in adenocarcinoma histology and never smokers (87). Patients with *HER2* mutant NSCLC have worse OS if treated without HER2 targeted therapy (88). Although there has not been any HER 2 targeted agent approved by NSCLC by the FDA, several agents have showed promising activity. Ado-trastuzumab emtansine, a HER2-targeted antibody-drug conjugate was found to be associated with an ORR of 44% in NSCLC with *HER2* exon 20 insertions and point mutations (89), and another HER2-targeted antibody-drug conjugate, trastuzumab deruxtecan, also showed an encouraging ORR of 55% in patients with metastatic HER2-overexpressing or *HER2*-mutant NSCLC whose disease had relapsed during standard treatment or was refractory to standard treatment (90). Both agents are included as novel therapeutic options for *HER2* mutant NSCLC in the current NCCN guidelines (2). Poziotinib, a tyrosine kinase inhibitor targeting *EGFR/HER2* exon 20 insertion mutation, was found to have an ORR of 27% in HER2 exon 20 mutant NSCLC, gaining fast track designation by FDA (91, 92).

The efficacy of immunotherapy in patients whose cancer harbors *HER2* mutation is largely unknown. The ORR associated with ICI among the 29 patients with exon 20 activating mutations in the IMMUNOTARGET study was only 7%. PFS was 2.5 months, and the 12-month PFS was 13.6 months. The OS was 20.3 months (31). The ORR among the 23 patients with exon 20 insertions included in the IMAD2 study by the French Lung Cancer Group was 27.3%. PFS was similar to the findings in the IMMUNOTARGET study and was 2.2 months, and the 12-month PFS was 22.9%. The mOS was an encouraging 20.4 months (47) (**Table 8**).

**Table 8:** Efficacy of ICIs in NSCLS with *HER2* mutations.

Reference	Characteristics	ORR, %	mPFS, months	mOS, months since start of ICI
Mazieres J., et al. (31)	*HER2* (n=29)	7%	2.9 (1^st^-3^rd^ line ICI)	20.3
			2.0 (>3^rd^ line)	
Guisier F., et al. (47)	*HER2* (n=23), number of lines prior to ICI =one median	27.3%	2.2	20.4

ICIs, immune checkpoint inhibitors; NSCLC, non-small cell lung cancer; ORR, overall response rate; mPFS, median progression-free survival; mOS, median overall survival.

## Discussion

To delineate the benefit of ICI treatment in NSCLC harboring actional mutations other than *EGFR* alterations, we reviewed the current available data in this area. We found that the ORR, median PFS, and OS with ICPi varied significantly across genetic alteration subgroups. While the ORR observed in the *BRAF*, *c-MET*, and *KRAS* altered NSCLC appeared to be similar to what had been observed in the non-selected NSCLC groups, the ORRs in the *ALK* and *RET* altered NSCLC groups were much lower (2).

Unlike *ALK* and *RET* fusions, *BRAF*, *MET*, and *KRAS* mutations can be seen in both smokers and non-smokers. The higher prevalence of smoking history in these patients could be a potential reason of the higher response rates since smoking has been found to be associated with the benefit derived from ICI treatment in some of the literatures (93, 94), although not confirmed by other studies (95). Other known predictive biomarkers for ICI treatment include PD-L1 expression level, microsatellite instability-high (MSI-H) or mismatch repair deficient (dMMR), and TMB (96–98). A higher percentage of PD-L1 TPS high (67%) was reported in *BRAF* non V600E mutant and *MET* mutant NSCLC in some of the reports (46), and a relatively higher response was seen in patients with PD-L1 TPS high *BRAF* mutant NSCLC (43), albeit the sample numbers was too small to draw any firm conclusion.

The current NCCN guidelines support the use of targeted therapy in the first line setting for advanced NSCLC with actionable genomic alterations involving *EGFR*, *ALK*, *ROS1*, *BRAF*, *NTRK1/2/3*, *METex14* skipping, and *RET.* After disease progression, chemoimmunotherapy is recommended for this population based on the guidelines (2). Although most of the chemoimmunotherapy trials excluded the *EGFR* and *ALK* altered NSCLC patients, these groups of patients were evaluated in the IMpower150 study if they have had progression with or unacceptable side effects from treatment with at least one approved tyrosine kinase inhibitor. The data showed improved PFS associated with the additional of atezolizumab to the bevacizumab, carboplatin, and paclitaxel combination in *EGFR* and *ALK* altered NSCLC (9.7 months vs. 6.1 months), providing direct evidence supporting the use of the regimen in this population (38). In our review, while not robust, we see that ICI’s do have activity in patients with NSCLC harbouring actionable mutations, and that a response can be seen after progression on targeted therapy, supporting offering chemoimmunotherapy in the post-targeted therapy setting. For *KRAS* G12C NSCLC, ICI monotherapy or chemoimmunotherapy is the current standard of care first line treatment. The data included in this review did confirm the benefits of ICI in the *KRAS* mutant NSCLC, supporting the current treatment approach. The benefit of ICI monotherapy in PD-L1 positive *KRAS* mutant NSLCL was suggested in retrospective studies, warranting further investigation on the selection of ICI monotherapy vs chemoimmunotherapy in this population (78, 79). Furthermore, data from prospective studies will be helpful to identify the best treatment sequence among targeted therapy, ICI, and chemotherapy.

Even in the subgroups where the benefits of ICIs were observed, the ORRs tended to be low. Therefore, developing predictive biomarkers for ICI therapy would be of great importance.

Co-occurring genomic alterations have been reported to be related to responses to immunotherapy through altering the microenvironment. For example, *LKB1/STK11* genomic alterations, a frequent co-occurring mutation in of *KRAS* mutant NSCLC, have been found to be associates with “immune-inert” state (99). This was supported by several studies including a retrospective study conducted in 103 NSCLC patients receiving ICIs. In this study, among the patient with *KRAS* mutations, the presence of concurrent *STK11* mutation or *STK11/TP53* mutations were associated with worse survival with ICI therapy. This association was not observed with chemotherapy, supporting the predictive roles of these co-mutations for ICI therapy in *KRAS* mutant NSCLC (15). The data from a retrospective analysis suggested that co-occurring *LKB1/STK11* mutations in *KRAS* mutant NSCLC may predict lower ORR, while data from another group showed no PFS or OS differences with or without concurrent mutant *LKB1/STK11* and/or mutant *KEPA1* in patients receiving combined chemoimmunotherapy and angiogenesis targeted agent (13, 81), raising the question whether angiogenesis targeted agent may help to overcome the challenge of the immune-inert state. Other co-occurring genomic alterations such as *P53, KEAP1, ATM, PTEN, CDKN2A* are common among *KRAS*-mutant NSCLC, and may play a role in determining response to ICI (100). Furthermore, a recent study showed that co-occurring mutations such as NOTCH and HR pathways were also found to be associated with increased efficacy of immunotherapy in advanced NSCLC (101). Therefore, identifying co-occurring mutations that are responsible for ICI response or resistance could potentially help to identify the candidate for ICI treatments and warrants further investigation in this group of patients.

How to overcome the resistance to ICI therapy is another great challenge. The mechanism of resistance is complex and is a combination of tumor-intrinsic and extrinsic factors. Many factors such as immune contexture and tumor microenvironment, expression of PD-L1 and LAG3, TMB, genetic and epigenetic alterations, antigen-presenting molecules (MHC, HLA) and microbiota may all contribute to the resistance to immunotherapy (102). The tumors with higher initial mutational burdens have been found to be associated with higher sensitivity to ICIs in some studies, although this association may be negated by other factors such as intratumoral heterogeneity and mutations (103). *RET* fusion positive NSCLC was found to have poor response to ICIs, and the alterations appears to be associated with lower TMB. In the analysis by Offin M. et al., the median TMB of *RET* altered NSCLC was significantly lower than that of the *RET* wild-type NSCLCs (1.75 versus 5.27 mutations/Mb, P <.0001) (61). The best outcome in patients in this study was stable disease which only lasted 5.6 months. In the report by Dudnik E. et al, the TBM was low in all 13 patients except one patient who had intermediate TMB, and the ORR in this report was also 0% (46). Nevertheless, an ORR of 37.5% was found among the nine evaluable patients reported by Guisier F. et al. Unfortunately, the TMB information was not available in this study, and it was unclear if the treatments were ICI monotherapy or chemoimmunotherapy. A prospective study to allow uniform treatment and collection of information on biomarkers such as TMB, PD-L1, MSI/MMR, tumor-infiltrating lymphocytes, whole-exome sequencing analysis on tumor samples and intestinal microbiome composition may be helpful to identify the resistance mechanisms.

*ALK* fusion positive NSCLC showed poor response to ICI in retrospective studies. However, this group of patients did benefit from ICI in the IMpower 150 trial, raising the question if the inhibition of angiogenesis could sensitize cancer cells to ICI therapy. Tumor angiogenesis can lead to immunosuppression through various mechanisms including maintaining an acidic/hypoxic and immunosuppressive environment, development of dysfunctional blood vessels which limits T cell trafficking, and suppression of dendritic cell maturation. Moreover, the angiogenic factors such as VEGF are also immunosuppressive (104). Therefore, further investigation is warranted in co-inhibition of angiogenic factors in NSCLC harboring actionable driver mutations undergoing ICI treatment.

With regard to the combination of ICIs and targeted therapies, a number of studies had evaluated the combiantion of *ALK* TKIs and different ICIs in NSCLC, including the combination of nivolumab with ceritinib or crizotinib and the combiantion of alectinib plus atezolizumab (105–107). However, significant toxicities were observed without survival benefit. In addition, there has been some concerning safety signals where ICI treatment is followed with targeted therapy (108). Reports showed risk of hepatotoxicity in a series of patients with *ALK*, *ROS1*, or *MET* exon 14 alterations who received ICI before crizotinib. Among the eleven patients treated with crizotinib following ICI, five patients (45.5%) developed grade 3 or 4 hepatotoxicity, whereas only 8% of those patients who received crizotinib alone experienced hepatotoxicity. The increased hepatotoxicity in sequentially treated patients led to permanent discontinuation of crizotinib in four of the five patients (108), highlighting the importance of establishing the presence of actionable mutations prior to initiating ICI therapy in patients with advanced NSCLC. The frequency and severity of toxicities associates with sequential use of ICI followed by targeted therapy may vary among different therapeutic agents. In the CodeBreaK100 phase II study evaluating Sotorasib in the beyond first-line setting, even though 91.3% patients had received ICI treatment prior to Sotorasib, the tolerability remained acceptable. Ongoing clinical trials DESTINY-Lung03 (NCT04686305) and the HUDSON trial (NCT03334617) are investigating the combination of T-DXd with immunotherapy, chemotherapy, novel anticancer agents and will hence shed more light on the approach in *HER2* mutant subgroup NSCLC patients.

Our review certainly has limitations. We were unable to comment on the response of *HER2*, *ROS1* and *NTRK* altered NSCLC to ICI as there were few reports in the literature, and the patient numbers in these reports were often very small. The challenges are obviously associated with the low incidences of these alterations. A recent report from Negrao et al. showed that *RET*, *ROS1* and *ALK* alterations were associated with low sensitivity to ICIs. However, there were only three *ROS1* fusion NSCLC patients included in the study, and the outcome of all three alterations were reported collectively (15). Furthermore, we were also unable to compare the responses to ICIs among different alterations which can be better investigated in prospective studies. Moreover, many studies included in this reivew did not have the biomarker information on all the evaluable patients. The ICI treatments and the number of lines of treament received previously by the patients also varied significantly. Additionally, it was not always clear whether the ICI treament was given as a monotherapy or in combination with cytotoxic chemotherapy. Randomized prospective studies would undoubtly provide more definitive information on this topic.

In conclusion, we see low responses to ICI in *ALK and RET* altered NSCLCs whereas *BRAF, KRAS and c-MET alterations* were associated with benefit from ICIs, and PD-L1 positive *KRAS* mutant NSCLCs may be more responsive to ICI monotherapy. Furthermore, the response to ICIs in *KRAS* mutant NSCLCs may vary depending on co-existing mutations, and responses to ICIs in *HER2*, *ROS1* and *NTRK* altered NSCLCs are less clear and varies significantly across a small number of studies. Ultimately, immunotherapy in the second line after progression on targeted agents can be considered as a treatment option at the discretion of treating physicians, following a mutual discussion with patients about the pros and cons of this approach.

## Author Contributions

All authors contributed equally to the writing, development, editing, and information gathering of the manuscript.

## Conflict of Interest

The authors declare that the research was conducted in the absence of any commercial or financial relationships that could be construed as a potential conflict of interest.

## Publisher’s Note

All claims expressed in this article are solely those of the authors and do not necessarily represent those of their affiliated organizations, or those of the publisher, the editors and the reviewers. Any product that may be evaluated in this article, or claim that may be made by its manufacturer, is not guaranteed or endorsed by the publisher.
